# An insight to PDAC tumor heterogeneity across pancreatic subregions using computed tomography images

**DOI:** 10.3389/fonc.2024.1378691

**Published:** 2024-11-12

**Authors:** Sehrish Javed, Touseef Ahmad Qureshi, Lixia Wang, Linda Azab, Srinivas Gaddam, Stephen J. Pandol, Debiao Li

**Affiliations:** Cedars Sinai Medical Center, Los Angeles, CA, United States

**Keywords:** pancreatic ductal adenocarcinoma (PDAC), tumor heterogeneity, pancreatic subregions, radiomics, pancreas cancer

## Abstract

Pancreatic Ductal Adenocarcinoma (PDAC) is an exceptionally deadly form of pancreatic cancer with an extremely low survival rate. From diagnosis to treatment, PDAC is highly challenging to manage. Studies have demonstrated that PDAC tumors in distinct regions of the pancreas exhibit unique characteristics, influencing symptoms, treatment responses, and survival rates. Gaining insight into the heterogeneity of PDAC tumors based on their location in the pancreas can significantly enhance overall management of PDAC. Previous studies have explored PDAC tumor heterogeneity across pancreatic subregions based on their genetic and molecular profiles through biopsy-based histologic assessment. However, biopsy examinations are highly invasive and impractical for large populations. Abdominal imaging, such as Computed Tomography (CT) offers a completely non-invasive means to evaluate PDAC tumor heterogeneity across pancreatic subregions and an opportunity to correlate image feature of tumors with treatment outcome and monitoring. In this study, we explored the inter-tumor heterogeneity in PDAC tumors across three primary pancreatic subregions: the head, body, and tail. Utilizing contrast-enhanced abdominal CT scans and a thorough radiomic analysis of PDAC tumors, several morphological and textural tumor features were identified to be notably different between tumors in the head and those in the body and tail regions. To validate the significance of the identified features, a machine learning ML model was trained to automatically classify PDAC tumors into their respective regions i.e. head or body/tail subregion using their CT features. The study involved 200 CT abdominal scans, with 100 used for radiomic analysis and model training, and the remaining 100 for model testing. The ML model achieved an average classification accuracy, sensitivity, and specificity of 87%, 86%, and 88% on the testing scans respectively. Evaluating the heterogeneity of PDAC tumors across pancreatic subregions provides valuable insights into tumor composition and has the potential to enhance diagnosis and personalize treatment based on tumor characteristics and location.

## 1 Introduction

Pancreatic Ductal Adenocarcinoma (PDAC) is a fatal disease, constituting over majority of pancreatic cancer cases (1). It currently ranks fourth among cancer-related causes of death and is expected to elevate to second by 2030 (2). A critical obstacle in managing PDAC lies in the limited understanding of PDAC tumor heterogeneity, including the histopathological, genetical, molecular, and spatial diversity within the pancreas (3). Ongoing research emphasizes the potential advantages of understanding such variations, offering opportunities for improved early detection, treatment effectiveness, and overall outcomes (4). Insight to PDAC tumor heterogeneity can potentially assist in reducing the risk of overlooking subtle tumors, misinterpreting suspicious lesions, unnecessary invasive procedures, and assisting in tailoring therapies aligned with tumor characteristics and location (4–6).

Research indicates that PDAC tumors exhibit variations across pancreatic subregions (head, body, and tail), including differences in histology, genomics, aggressiveness, symptoms, and treatment responsiveness (7, 8). For instance, head tumors display less aggressiveness, manifest weight loss, and respond better to Gemcitabine, while body/tail tumors cause abdominal pain and favor Fluorouracil (9–11). These studies also propose PDAC characterization, leading to the identification of distinct tumor subtypes. These subtypes with variable overall survival, tumor growth, therapy response, and patient prognosis have been associated with the location of tumors in the pancreas. These distinctions lead to varying incidence rates (71%, 13%, 16%), metastasis (42%, 68%, 84%), 2-year survival (44%, 27%, 27%), and resection rates (17%, 4%, 7%) for head, body, and tail respectively (12, 13).

Considering these facts, research strongly indicates that characterizing tumor heterogeneity can significantly enhance precision medicine methods and improve the overall management of PDAC. Histologic assessment allows for a precise analysis of PDAC tumor heterogeneity using tissue samples obtained through biopsy. However, PDAC biopsy examinations are highly invasive, impractical for large populations, and cannot be conducted frequently. Moreover, in the event of sampling errors, PDAC biopsy fails to comprehensively comprehend the spatial condition of the tumor, thereby limiting the ability to fully capture the tumor’s complexity (14, 15).

Noninvasive techniques, such as imaging, have demonstrated to be a secure and effective means of understanding the tumor microenvironment. Imaging provides excellent spatial resolution and the capacity to evaluate the overall heterogeneity of the tumor. Furthermore, it is minimally invasive or noninvasive, enabling repeated examinations and coverage of multiple tumor sites. Imaging has played a vital role in identifying unique tumor patterns associated with their genomic composition and specific positions within organs (16). Numerous studies have investigated the spatial heterogeneity of tumors in various organs, including esophageal, lung, colorectal, liver, breast, head and neck (17–21), irrespective of their underlying association with genetic and molecular profiling, using computed tomography (CT) scans. These studies have linked distinct tumor patterns to predict tumor response, treatment outcome, staging, recurrence, and overall survival. Unfortunately, no study has explored the heterogeneity of PDAC tumors concerning their location in imaging, leaving a significant research question unexplored that holds high clinical value.

This exploratory pilot study provides insights into the inter-tumor heterogeneity of PDAC across different pancreatic subregions using contrast-enhanced abdominal CT scans. The investigation involves a comprehensive radiomic analysis, focusing on morphological and textural features of tumors identified in distinct pancreas subregions. The results demonstrate significant differences in various CT features between tumors located in the pancreatic head and those in the body and tail regions. These findings support previous research highlighting genomic disparities among pancreatic head tumors and those in other regions, paving the way for further noninvasive studies linking subregional PDAC tumor heterogeneity to various clinical outcomes. Additionally, a machine learning (ML) model was trained to autonomously categorize PDAC tumors into their respective subregions (head or body/tail) based on their distinct CT features. External validation of the model yielded highly satisfactory results, reinforcing the potential of this study’s findings and suggesting further validation on larger datasets. Such a model holds promise for applications including precise detection of PDAC tumors in CT images.The clinical endpoint of this study is to investigate if there are image features of pancreas tumors that are distinctive in head vs body/tumors. This research study deduced a proof of concept that image-based differences exist in these tumors. However, correlating the image-based differences in head vs body/tail tumors with the differences identified by the histology and molecular profile was not explored in this study, and this is the future research plan.

## 2 Materials and methods

In the context of PDAC management, particularly for screening purposes, abdominal CT plays a pivotal role (22). Abdominal CT is the established standard protocol and the primary, widely accepted initial radiologic modality for evaluating patients with PDAC (23). Examining CT for evaluating PDAC tumor heterogeneity provides an additional dimension to existing CT-based imaging biomarkers. Traditionally, these biomarkers have primarily focused on quantifying tumor size, attenuation, and perfusion. By extending the application of CT to assess tumor heterogeneity across pancreatic subregions, the study gains insights into previously unexplored aspects of PDAC. Hence, examining CT scans in this research is strongly justified, as it aligns with the well-established role of abdominal CT in PDAC management and offers valuable insights into tumor heterogeneity, complementing the existing array of imaging biomarkers.

A large dataset comprising of the 200 contrast-enhanced abdominal CT scans was obtained from the host institution. Each scan originates from an individual with a confirmed PDAC diagnosis, verified through biopsy procedures and displaying evident tumor presence in the scan. These tumors are situated in various subregions within the pancreas, consisting of 116 and 84 tumors located in the head and body/tail regions respectively. The precise localization was verified through a thorough examination of the subjects’ pathological reports and by manually labeling the tumors, as elaborated upon later. Also, the PDAC cases included in the dataset span different cancer stages from T1-T4. Furthermore, all scans selected for analysis were obtained during the venous phase of CT imaging, chosen for its superior tumor margin visibility compared to other phases. Each scan is characterized by a 16-bit depth and a slice resolution of 512 by 512 along the x- and y-axes, with variable resolution along the z-axis.

Moreover, the 200 cases were evenly distributed into two subsets, namely *D*_1_ (for preliminary analysis and model training) and *D*_2_ (model validation). These subsets comprised of randomly selected scans, with head tumor in 58 scans for each of *D*_1_ and *D*_2_, and body/tail tumors in 42 scans for *D*_1_ and *D*_2_. The demographics information of 200 cases is provided in **Table 1**.

**Table 1:** Patients data summary.

Demographics	Patients	
Gender	Male (146)	Female(54)
Mean Age	40-50 years (29)	50-75 years (171)
Tumor site	Head tumors (116)	Body/tail tumors (84)
Tumor stage	TNM stage I-II (47)	TNM stage III-IV (153)

Table shows gender, ages, tumor sites, tumor stages for patients.

In adherence to rigorous privacy protocols, all scans were anonymized prior to transferring to the host institute and performing analysis. As all the data obtained for this study is retrospective, informed consent was not a requirement for inclusion within the study cohort.

### 2.1 Data labeling and preprocessing

Precisely delineating pancreatic tumors necessitates a detailed understanding of the pancreas, its intricate subregions, and the tumors themselves. Given the complexity of pancreatic anatomy, manual delineation is a challenging process (24). To ensure the utmost accuracy and consistency, a detailed multistage labeling procedure was performed for each of these structures.

The pancreas, situated transversely in the posterior region of the abdomen, is visible in the axial view of abdominal CT scans. Initially, an automated technique (25) specifically designed for pancreas segmentation in CT images was employed to establish the initial boundaries of the pancreas across all scans in *D*_1_ and *D*_2_. Subsequently, these labels underwent thorough evaluation and, if necessary, correction by two research associates. Finally, the accuracy of these labels was validated with consensus by two highly experienced radiologists, each possessing several years of expertise at the host institute.

The pancreatic head region exhibits a slightly flattened morphology, occupying a central position along the duodenum’s curvature. The pancreas neck is approximately 2 cm long and is commonly included as part of the head. Previous studies focusing on segmentation of pancreatic subregions have considered pancreatic neck as part of pancreatic head (26). Adjacent to this region is the body subregion, which gradually tapers into the tail subregion. In terms of size, the pancreas typically possesses anteroposterior diameters ranging from 1 to 3 centimeters and lengths ranging from 12 to 15 centimeters. These dimensions correspond to the head, body, and tail regions, constituting approximately 40%, 33%, and 26% of the total pancreas, respectively. Utilizing the segmented pancreas images, these three subregions across all scans in *D*_1_ and *D*_2_ were delineated using an existing automated technique (27). To ensure accuracy and labeling consistency, the same two validation steps were implemented, mirroring the process employed for pancreas segmentation.

**Figure 1:**
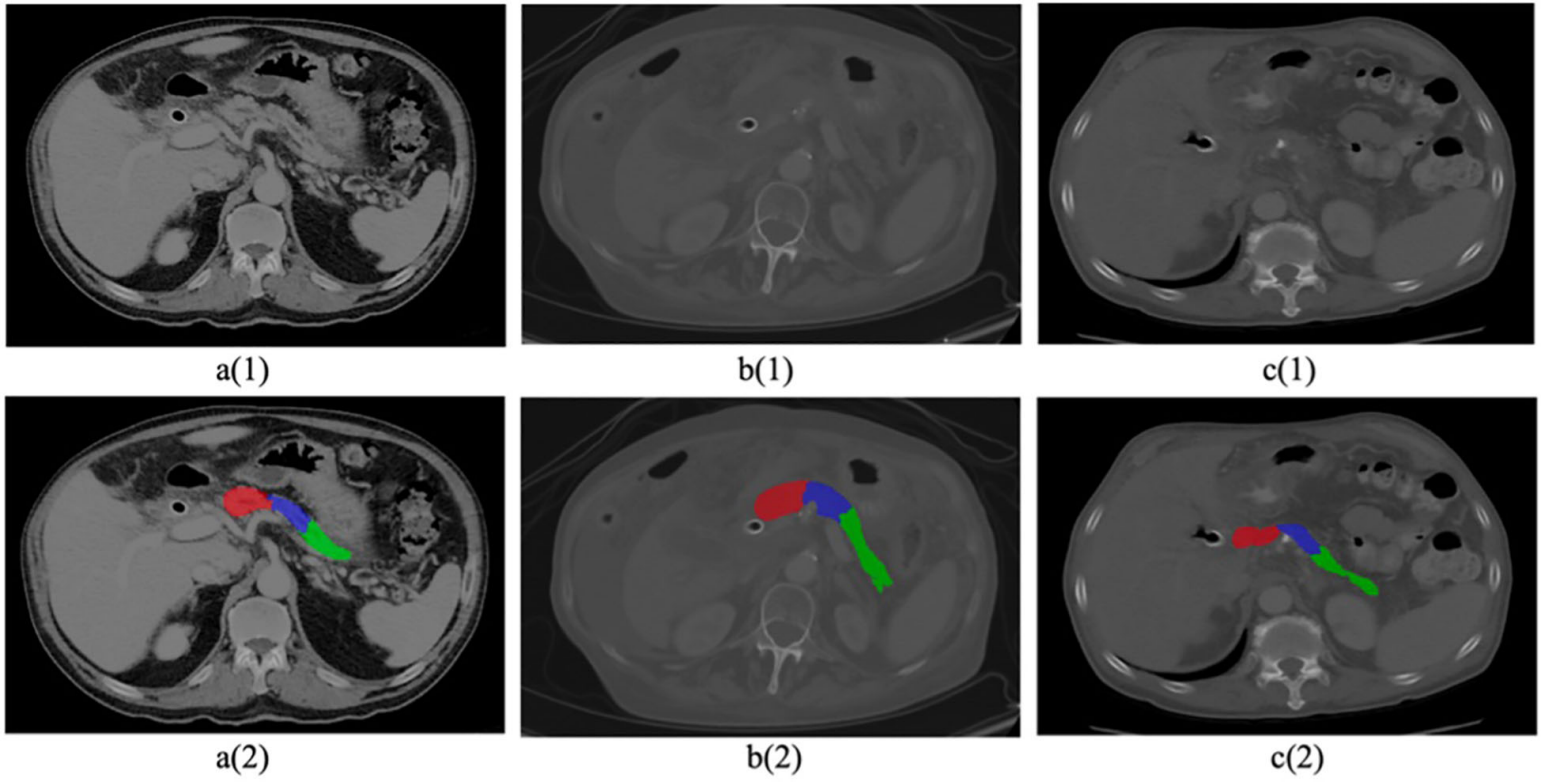
Sample CT images with cancerous pancreas. Top row 1 (a–c) shows original raw images, Bottom row 2 (a–c) shows segmented pancreas and subregions (head in red, body in blue, tail in green).

Finally, the tumors within all scans from both *D*_1_ and *D*_2_ were segmented manually by our research associates. We aimed to minimize any potential inter-reader variability which yielded 86% consistency in labeling outcomes. Subsequently, the initial labels underwent independent validation by our two experienced radiologists at the host institute. To further enhance precision, both radiologists reviewed each other’s delineations, resulting in a notable 98% overlap in labeling outcomes. The remaining 2% of labeling discrepancies were effectively addressed through collaborative discussions between the two radiologists, ultimately achieving a harmonious consensus. The mean volume of tumors were calculated by the radiologists during the manual labelling process. The mean volume of the head tumors ranged between 2.4cm to 3.1cm, while for body/tail tumors ranged between 5.6cm to 6.1cm. A representative delineation of these three structures is provided in **Figure 1**.

Furthermore, no preprocessing methods were employed on the scans, except for signal intensity normalization, which was scaled to the range of 0 to 1.

## 3 Results

### 3.1 PDAC tumor heterogeneity across pancreatic subregions

**Figure 2:**
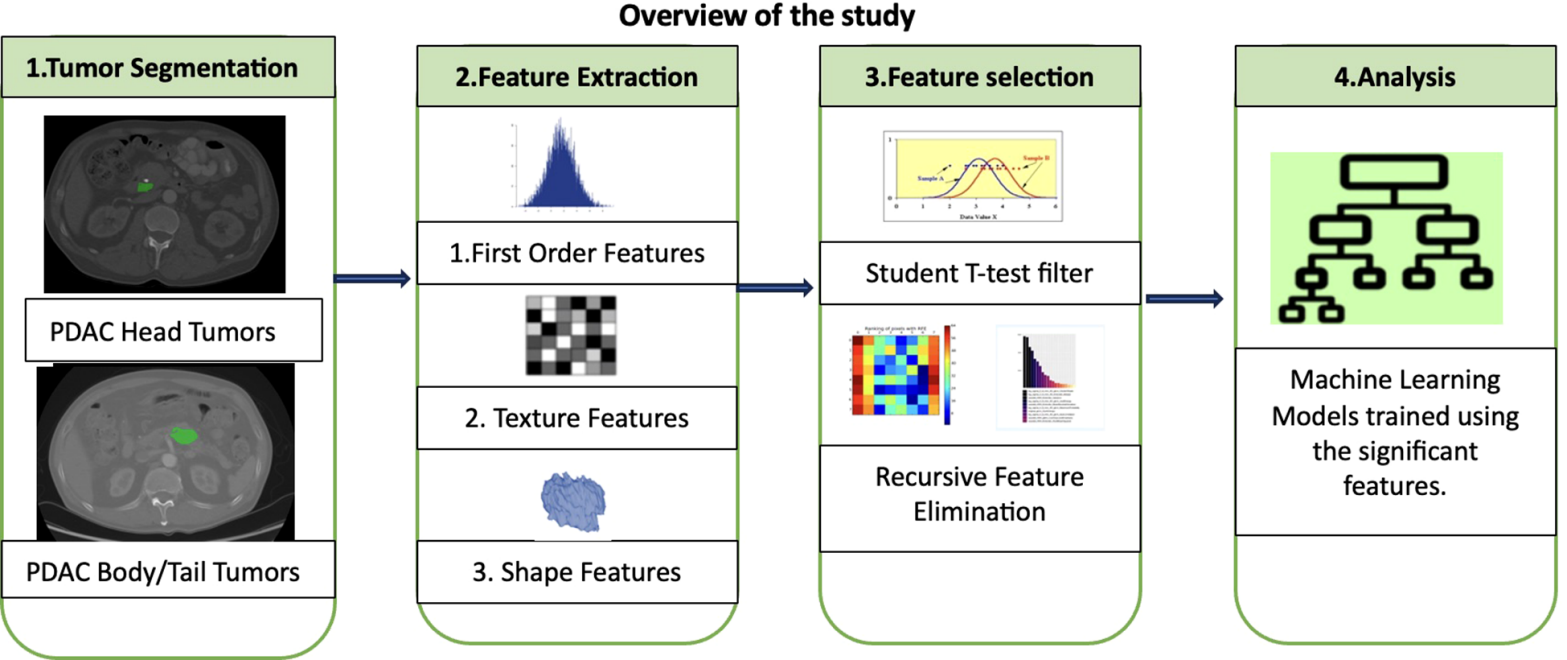
Overview of the study.

A comprehensive investigation into the heterogeneity of PDAC tumors across different pancreatic subregions using radiomics was conducted. This approach involved analyzing both the morphological and textural characteristics of the identified tumors in CT images. Notably, the tumors in the body and tail together were grouped as a single category for clinical relevance (28). The study hypothesis was that PDAC tumors in the head would display distinct patterns in terms of shape, size, and signal intensity compared to those in the body and tail. Once the significant features were identified, an ML classifier to automate the categorization of PDAC tumors into their respective groups was developed. The subsequent sections provide a detailed explanation of the radiomic analysis and model development. **Figure 2** shows an overview of the entire study including major steps in radiomic analysis and ML modelling.

### 3.2 Radiomic Analysis of PDAC Tumors

A comprehensive array of radiomic features from every tumor instance within all 100 CT scans found in dataset *D*_1_ was extracted. This yielded two distinct feature sets, with each set specifically designed for its respective class. Each individual feature served as a unique, quantifiable descriptor of the tumor, providing valuable insights into the spatial correlations among adjacent voxels within predefined proximities. To assign numerical values to these features, an evaluation of signal intensities across all voxels within the tumor region was conducted, spanning all slices within a given scan.

An important aspect of radiomic analysis involves the rigorous examination of feature variations, which are contingent on the interplay of three key radiomic parameters: *bin* size, *kernel* size, and *angle* (29). The *bin* size was instrumental in discretizing continuous voxel values within CT images into equivalent bins. This approach was employed to avoid unwarranted differentiation of pixels with closely clustered signal intensities. For instance, a minimal value difference of 0.01 between two adjacent voxels within the tumor region likely results from noise and does not convey meaningful information about spatial heterogeneity. The *kernel*, represented as a square convolution matrix, is defined as a specific area *A* surrounding a voxel *x*. Within this defined region, comprehensive calculations of spatial relationships with neighboring voxels were conducted. The *angle* parameter played a significant role in determining directions during the assessment of associations between voxel *x* and its neighboring voxels within the area *A*. Throughout the feature extraction process, the *bin* size ranged from 2^1^ to 2^8^, the *kernel* window size varied from 1 to 5, and the *angle* was considered in all four quadrants.

Each radiomic feature represented a fundamental tumor characteristic, incorporating dimensions such as shape, size, texture, and signal intensity. These attributes were quantified as individual numerical values using predefined mathematical formulas. In this comprehensive collection of features, the entire tumor was treated as a unified ‘region of interest.’ For example, to measure signal intensity within the tumor, the mean grey level values of all voxels enclosed by the tumor boundary across all slices of a three-dimensional CT scan were computed. A variety of radiomic features used in the analysis are detailed in **Table 2**.

**Table 2:** List of types of radiomic features with example features for each type.

Feature Type	Feature Examples	Total Features
First Order Statistics	Kurtosis, Percentiles, Range	15
Grey-level Co-occurrence Matrix	Cluster shade, Contrast, Autocorrelation	20
Grey Level Run Length Matrix	Run percentage, Run entropy	15
Grey Level Size Zone Matrix	Zone percentage, Zone variance	14
Grey Level Dependence Matrix	Small dependence emphasis	12
Shape-based Features 2D and 3D	Volume, Surface area, Sphericity	20
Additional features	Complexity, Busyness	5

With all conceivable combinations of the three parameters for each of the different types of features, the process yielded over 4000 radiomic features for each of 58 head tumors and 42 body/tail tumors using all images in *D*_1_. All features were extracted utilizing a custom-built radiomic feature extraction application.

Prior to conducting the analysis, all the extracted features underwent a filtering process to eliminate values that were unusable or infinite. Subsequently, a pairwise comparison of these features was performed to identify those that displayed significant differences between head tumors and body/tail tumors. Specifically, the features extracted from all head tumors in dataset *D*_1_ were systematically compared to those from all body/tail tumors in the same dataset using the statistical t-test. This comparison revealed that approximately 6% of the extracted features exhibited statistical significance at a *p*-value of 0.05, indicating their potential to distinguish between head and body/tail tumors. A Manhattan plot, provides *p*-values for all the features subjected to the significance test. To visually depict the differences in features across subregions, a limited selection of up to 5 randomly chosen significant features was integrated. These findings support the fundamental hypothesis concerning the distinct characteristics of tumors in various pancreatic subregions.

**Figure 3:**
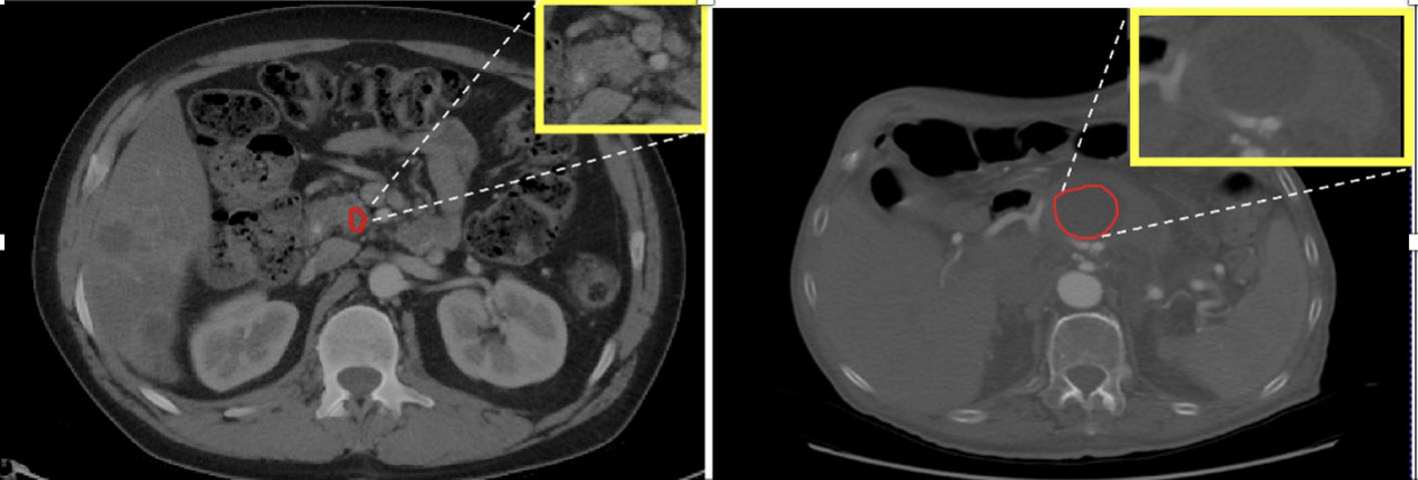
Left: PDAC tumor in the head appears hyperintense or bright. Right: PDAC tumor in the body appears less intense or dark with visible contrast between tumor and non-tumor area.

The significant features identified in the analysis primarily pertained to the textural characteristics of the tumor, which can be largely attributed to variations in the cellular arrangement within the tumor. Another notable difference between head and body/tail tumors was their size, with head tumors being significantly smaller than those in the body/tail. This discrepancy may be due to the inherently aggressive nature and faster growth of body/tail tumors, as observed in previous studies (30). Additionally, the mean signal intensities of head tumors were comparatively higher, resulting in a ‘hyperintense’ appearance, as depicted in **Figure 3**. One plausible explanation for this observation is that head tumors often exert pressure on adjacent structures, such as the pancreatic duct and bile duct, leading to disruptions in fluid flow and altered contrast levels (resulting in high intensity) on CT scans (30). A similar pattern was also observed in the body, which exhibited slightly higher CT signals compared to those in the tail. Furthermore, the shape of tumors across the three subregions was found to be insignificantly different. These findings from the analysis are promising and have spurred further validation efforts using ML algorithms to automate tumor characterization based on their detected region.

### 3.3 Classification of PDAC Tumors

The significant features identified in the radiomic analysis were subsequently utilized to train an automated binary classification system for PDAC tumors, categorizing them into two distinct classes: head tumors and body/tail tumors. Multiple ML methods were employed to train the classifier for binary tumor classification using the CT scans. Any instance in which the classifier erroneously categorizes a head tumor as a body/tail tumor or vice versa is documented as a misclassification.

Seven widely used ML classifiers, including Naïve Bayes, K-Nearest Neighbor, K-Means, Support Vector Machine, Linear Regression, Ensembled Bagged Trees, and Linear Discriminant were trained for binary classification tasks, accompanied by the Recursive Feature Elimination (RFE) technique (31). RFE was integrated into each classifier, systematically eliminating less crucial features by exploring various combinations of the identified predictors. The primary aim was to enhance training accuracy while adhering to the predefined classification criteria. Additionally, the RFE process was intricately fine-tuned to identify and retain a subset of a maximum of five features for each of the classifiers. This does not imply that each classifier must utilize the same set of features.

Rather, this strategic adjustment was made to prevent the risk of potential overfitting in any of the classifiers.

All seven classifiers were trained using dataset D_1_ and tested or validated using external dataset D_2._ Out of all these classifiers, the Naïve Bayes model achieved the highest testing accuracy of 86%, surpassing the other six classifiers. It effectively identified five standout features: Long-run low grey-level emphasis, Gaussian left polar, Inverse Gaussian left polar, Inverse cluster shade, and Inverse cluster tendency. These features were regarded as the most influential predictors, as their collective contribution led to the highest classification accuracy during validation.

The confusion matrix for Naïve Bayes model derived during validation process, showing the true positive vs predicted head and body/tail tumors is presented in **Table 3**.

**Table 3:** Confusion matrix for Naïve Bayes model showing the true and predicted head tumors and body/tail tumors.

	True Head Tumors	True Body/Tail Tumors
**Predicted Head Tumors**	50	5
**Predicted Body/Tail Tumors**	8	37

Green for correct classification. Red for wrong classification.

**Figure 4:**
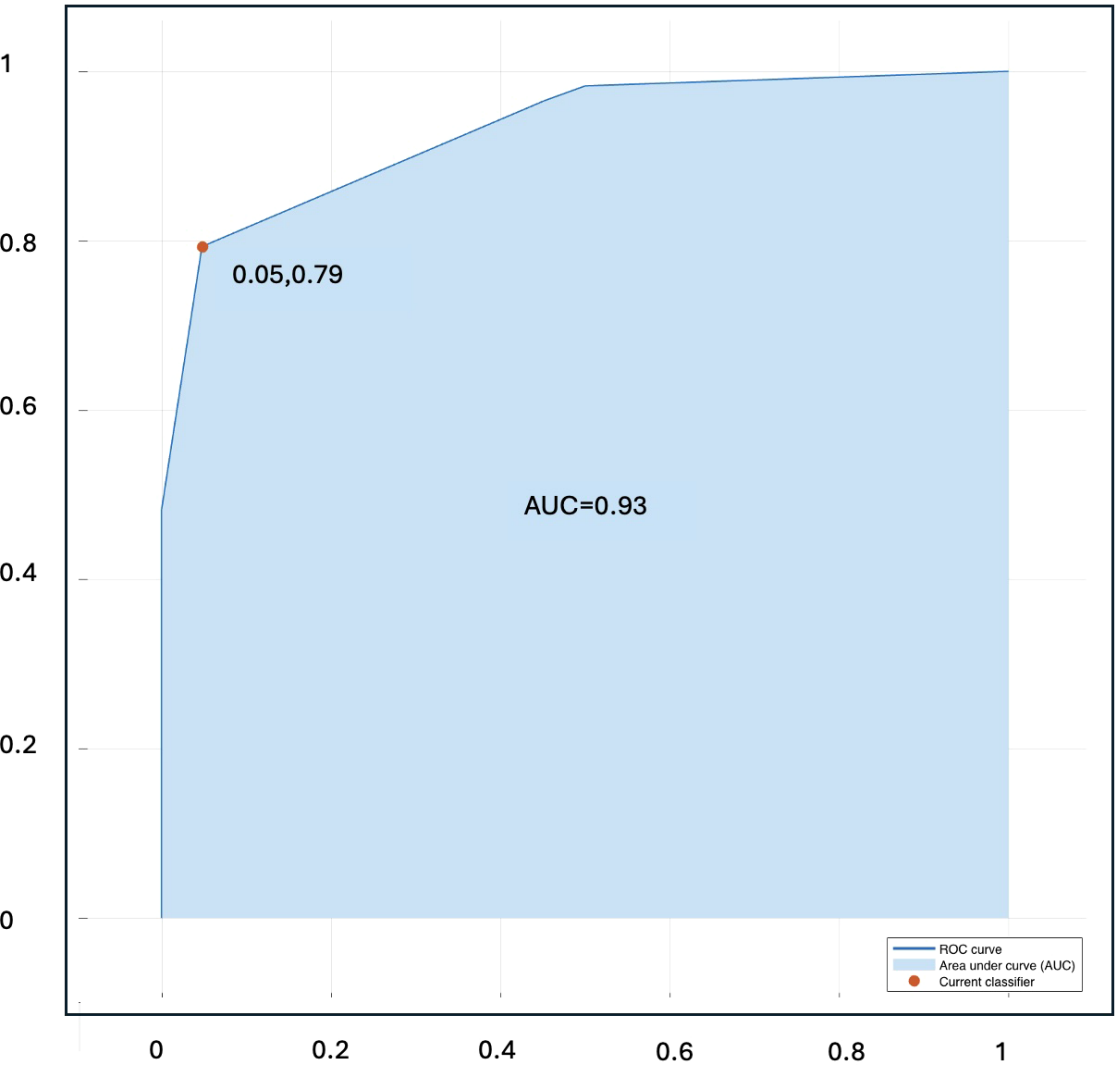
ROC for Naïve Bayes classifier showing classification performance with AUC=0.93.

**Figure 4** present ROC curve illustrating the classification performance of the Naïve Bayes classifier in the validation sets.

The obtained results from the automated classification are encouraging and substantiate the proof of concept regarding the effectiveness of utilizing CT imaging features for the efficient characterization of PDAC tumor heterogeneity across various pancreatic subregions. In most misclassification cases, it has been noticed that tumors located at the boundary between the head and body tend to be a common factor, suggesting that these cases might lead to model deviations.

## 4 Discussion

### Tumor heterogeneity across pancreatic subregions

Pancreatic Ductal Adenocarcinoma (PDAC) is a genetically complex and heterogeneous disease. The presence of heterogeneity in PDAC tumors is associated with various factors, such as differences in genomic subtypes, the expression of growth and angiogenic factors, and the characteristics of the tumor microenvironment (7–9). Additionally, studies have highlighted that this heterogeneity is closely linked to the distinct pancreatic subregion where the tumor is located. Moreover, variations in PDAC tumors across different subregions have been observed to manifest in significant differences in symptoms, metastasis patterns, and responses to treatment (10). Therefore, exploring and characterizing the heterogeneity of PDAC tumors based on their location holds significant clinical relevance.

Existing literature acknowledges numerous studies indicating that PDAC tumors located in different pancreatic subregions exhibit significantly distinct genetic and molecular profiles (7–9). Several models have been proposed to characterize the spatial heterogeneity of PDAC tumors based on genetic and molecular features. For instance, Collisson et al. (8) identified three tumor subtypes (classical, quasi-mesenchymal, exocrine-like), Moffitt et al. (9) distinguished two stroma subtypes (normal, activated) and two tumor subtypes (classical, basal-like), and Bailey et al. (7) categorized PDAC into four subtypes (squamous, pancreatic progenitor, immunogenic, aberrantly differentiated endocrine exocrine). These subtypes impact overall survival, tumor growth, therapy response, and patient prognosis, and are associated with the tumor’s location.

### Evaluating PDAC tumor heterogeneity via CT imaging

Histologic methods can be employed to assess PDAC heterogeneity across pancreatic subregions, providing precise evaluation and high spatial resolution of biopsy tissue samples. However, biopsies are invasive, challenging to obtain in some cases, prone to sampling errors due to the complex location of the pancreas in the abdomen, and can only sample a small and random portion of the entire tumor. Moreover, determining intratumor heterogeneity from a single biopsy has limitations, making it incomplete or potentially misleading. As an alternative, abdominal CT imaging offers an opportunity for a comprehensive evaluation of tumor heterogeneity in the pancreas. It provides excellent spatial resolution, is non-invasive, can be repeated, and covers multiple tumor sites. However, the assessment of PDAC tumor heterogeneity using CT imaging has been predominantly qualitative and lacks a standardized quantitative methodology. While other imaging techniques like Magnetic Resonance Imaging (MRI) may offer superior tissue contrast for PDAC tumors and provide deeper insights into tumor heterogeneity, CT is preferred due to its widespread use in screening for PDAC, especially in its early stages (23).

Also, the CT imaging has been extensively utilized in numerous studies investigating the spatial heterogeneity of tumors in various organs, including the lungs, head and neck (21), and breast (20). Findings obtained on tumor heterogeneity in different organs through radiomics analysis of CT images have been further correlated and verified with the histological findings, efficiently assisting in clinical decision during treatment planning and disease management (32). Furthermore, CT-based tumor heterogeneity has been used as a basis in many clinically relevant studies such as predicting treatment response in esophageal carcinoma (17), forecasting distant metastasis in lung adenocarcinomas (21), and differentiating between aggressive and nonaggressive malignant tumors (33).

### Study Findings

This first study aimed to investigate the morphological and textural heterogeneity of PDAC tumors in different pancreatic subregions using CT images and identify features specifically associated with tumors in distinct subregions. Based on an extensive radiomic analysis of PDAC tumors, the study demonstrated a significant statistical difference in CT texture between PDAC tumors located in the head and those in the body/tail, consistent with findings from previous non-imaging studies on PDAC tumor heterogeneity. To assess the discriminative utility of the identified features, various traditional machine learning algorithms were trained to automatically categorize PDAC tumors in CT images into their regional class (head, body/tail). The analysis and model development were performed on a large dataset of 200 contrast-enhanced venous-phase CT scans. Both the analysis findings and the model results were stable and satisfactory.

### Scope of the study

Examining PDAC tumors across various pancreatic subregions through radiomic analysis of abdominal CT provide insights into the heterogeneity of these tumors, assist in leading to targeted therapies and improved treatment strategies for patients with tumors in specific subregions. Although various techniques exist for quantifying tumor heterogeneity in CT imaging analysis, some of these derive features from histograms, such as percentile values, standard deviation (SD), and enhancing fraction. These metrics might not account for the spatial distribution of intensity values. Conversely, radiomics, as proposed in this study, take spatial information into consideration, and offer supplementary information, including average signal intensity, beyond what histogram-derived measures provide. Radiologists may visually perceive some features, while others are more abstract.

The current study carries substantial clinical importance, with potential applications including the advancement of a more sophisticated PDAC tumor detection model in CT images. This model could adaptively choose morphological and textural features based on the specific subregion, addressing the existing issue of mis-detecting small tumors in the early stage. Additionally, comprehending PDAC tumor heterogeneity has the potential to refine the assessment of suspected pancreatic lesions, reducing misinterpretations caused by PDAC mimics or other conditions like IPMNs (Intraductal Papillary Mucinous Neoplasms) (34), which currently pose challenges to accurate detection. Furthermore, exploring PDAC tumor heterogeneity concerning its location could contribute to predicting PDAC survival outcomes, in contrast to current studies that either overlook the spatial heterogeneity of tumors (35) or focus solely on limited regions (such as the head region) (36).

### Study limitations and future work

The study is confined to observing the spatial distribution heterogeneity of tumors across pancreatic subregions on CT imaging and does not correlate it with the underlying biological factors influencing this variation. The primary goal of the study was only to demonstrate the quantifiability of PDAC tumor heterogeneity across subregions on CT images through extensive radiomic analysis. Nevertheless, the study lays the groundwork for potential future research to explore correlations between this heterogeneity and genomic sequences and molecular profiles, offering deeper insights. Additionally, the study does not account for the clinical stage of PDAC when assessing tumor heterogeneity, which can significantly impact various tumor characteristics such as size, shape, and texture, reflecting clonal evolution over time. Furthermore, the study exclusively focuses on inter-tumor heterogeneity across different subregions within tumors and does not investigate intra-tumor variations within a specific subregion.

The primary aim of this study is to provide a proof the concept and encourage researchers to further investigate and validate the heterogeneity of PDAC tumors on large diverse datasets of CT images. In future investigations, we plan to consider the stage of PDAC and intra-tumor variations within each subregion to gain a more comprehensive understanding of PDAC tumor heterogeneity.

## 5 Conclusion

This first study aimed to explore potential heterogeneity among Pancreatic Ductal Adenocarcinoma (PDAC) tumors based on their locations within the pancreas as observed in Computed Tomography (CT) scans. A comprehensive radiomics analysis of PDAC tumors in pancreatic subregions (head, body, tail) revealed several statistically significant features distinguishing head tumors from those in the body and tail. Following this, a machine learning model was trained for binary classification, effectively distinguishing between head and body/tail tumors. The model exhibited satisfactory performance on a completely independent dataset, prompting further investigations on larger datasets. The quantification of tumor heterogeneity across pancreatic subregions through CT imaging holds promise for various clinically important applications, including improved detection and interpretation of pancreatic tumors.

## Data Availability

The data analyzed in this study is subject to the following licenses/restrictions: The dataset will be made publicly available after the completion of RO1 grant. Requests to access these datasets should be directed to Sehrish Javed, sehrish.javed@cshs.org.
